# Feature Selection Method Based on Partial Least Squares and Analysis of Traditional Chinese Medicine Data

**DOI:** 10.1155/2019/9580126

**Published:** 2019-07-01

**Authors:** Canyi Huang, Jianqiang Du, Bin Nie, Riyue Yu, Wangping Xiong, Qingxia Zeng

**Affiliations:** ^1^Computer School, Jiangxi University of Traditional Chinese Medicine, Nanchang 330004, China; ^2^College of Pharmacy, Jiangxi University of Traditional Chinese Medicine, Nanchang 330004, China

## Abstract

The partial least squares method has many advantages in multivariable linear regression, but it does not include the function of feature selection. This method cannot screen for the best feature subset (referred to in this study as the “Gold Standard”) or optimize the model, although contrarily using the L1 norm can achieve the sparse representation of parameters, leading to feature selection. In this study, a feature selection method based on partial least squares is proposed. In the new method, exploiting partial least squares allows extraction of the latent variables required for performing multivariable linear regression, and this method applies the L1 regular term constraint to the sum of the absolute values of the regression coefficients. This technique is then combined with the coordinate descent method to perform multiple iterations to select a better feature subset. Analyzing traditional Chinese medicine data and University of California, Irvine (UCI), datasets with the model, the experimental results show that the feature selection method based on partial least squares exhibits preferable adaptability for traditional Chinese medicine data and UCI datasets.

## 1. Introduction

In the era of rapid information technology development, data have become increasingly important. As one of the key techniques of data mining, statistical analysis methods have received extensive attention in the fields of biomedicine, physical chemistry, and traditional Chinese medicine [1–3]. One target variable, however, is often affected by other features, which exert different degrees of influence. Traditional Chinese medicine data with multicollinearity, meanwhile, include somewhat irrelevant as well as redundant information that not only increases the time and space complexity of the model but also seriously affects its accuracy and operational efficiency. In conventional statistical analysis methods, calculating the regression coefficients has the partial advantage of reflecting the relationships between features when dealing with such data [4, 5], whereas for data containing irrelevant and redundant features, feature selection may be achieved only minimally; in which case, we lose the chance to achieve model optimization and improved regression accuracy. Therefore, given the multicollinearity of traditional Chinese medicine data as well as the problem of irrelevant and redundant content, there is an urgent need to find a method of data analysis that can remove irrelevant and redundant features from the original dataset, thereby overcoming multicollinearity and screening out the “Gold Standard” feature subset in order to construct a robust model.

The remainder of this manuscript is organized as follows. Related research is introduced in Section 2.  In Section 3, the new model is described in detail. In Section 4, 3 traditional Chinese medicine datasets and 3 public UCI datasets are used in the new model and subjected to experimental analysis. The new model is compared with several existing algorithms in order to further verify its feasibility and effectiveness. The final section concludes this study with a brief summary and discussion.

## 2. Related Work

Feature selection, as an effective dimension reduction method, involves choosing a subset of features from the original set that has an acceptable distinguishing capability based on a particular standard [6, 7], thereby retaining the features that are most effective and favorable for regression (or classification) while decreasing the complexity of the algorithm. This method has attracted the attention of numerous researchers. In the medical field, for example, Peng et al. [8] have developed a multimodal feature selection method based on a hypergraph for multitask feature selection in order to choose effective brain region data; Ye et al. [9] have proposed an informative gene selection method based on symmetric uncertainty and support vector machine (SVM) recursive feature elimination that can effectively remove irrelevant genes; and Zhang et al. [10] proposed a hybrid feature selection algorithm that can select genetic subsets with strong classification ability. In addition, feature selection methods have successfully been applied in other fields. Hu et al. [11] proposed a feature selection algorithm for joint spectral clustering and neighborhood mutual information that can remove features unrelated to markers; Huang et al. [12] proposed a feature selection algorithm based on multilabel ReliefF that can remove irrelevant features while opting for features having strong correlations with categories. In terms of our research questions, most of the experimental data from traditional Chinese medicine present characteristics having multielement, multitarget, and strong multicollinearity problems [13]. Although the feature selection method can eliminate the irrelevant features in order to achieve an improved dimensionality reduction effect, this will not be a good solution to the multicollinearity problem. Therefore, we are in the process of pursuing further improvements and continuing our exploration.

As a nonparametric multivariate statistical analysis method, partial least squares (PLS) provides a regression modeling method for multidependent variables to multi-independent variables in order to effectively solve the problem of multicollinearity [14, 15] when there is a high correlation between independent variables. Based on preponderance, some researchers have proposed a series of improved models. You et al. [16] have proposed a feature selection method (PLSRFE) by combining PLS with the recursive feature elimination method (RFE). The PLSRFE can remove irrelevant features and select a small number of features, although it lacks reliability due to the default parameters applied. Shang et al. [17] presented a robust feature selection and classification algorithm based on partial least squares regression to solve the problem of multicollinearity and redundancy in features, yet the follow-up question as to whether the selection of parameters in the model is sufficient remains to be answered. Nagaraja et al. [18] connected partial least squares regression and optimization experimental design in order to select features by analyzing the model parameters while ignoring the interference of noise samples, which weakened the robustness of the model. In view of the shortcomings of the methods proposed above, by only using partial least squares for all data in the regression model without considering the advantages of the feature selection method, we create a model with poor interpretability and issues such as overfitting, shortcomings that make it impossible to achieve “Gold Standard” screening, and model optimization. Therefore, the goal of this study is to determine how to effectively perform feature selection on multicollinear traditional Chinese medicine (TCM) data and establish a regression model that is as simple and accurate as possible.

In a feature selection study, higher-quality feature selection methods should exhibit the following characteristics [19]: (1) interpretability, meaning that the features selected in the model have scientific significance; (2) acceptable model stability; (3) avoidance of deviations in the hypothesis test; and (4) model calculation complexity within a manageable range. Traditional feature selection methods such as stepwise regression, ridge regression, and principal component regression [20, 21] only satisfy some of the above characteristics. Therefore, effectively overcoming such problems and achieving better feature selection results have become a research point for regression and classification. In order to determine a feasible solution to this problem, Tibshirani has presented a feature selection method called “lasso” [22] and applied it successfully by taking inspiration from both the ridge regression algorithm proposed by You et al. [23] and the nonnegative garrote algorithm proposed by Breiman [24]. The lasso method compresses the regression coefficients by using the absolute value function of the model coefficients as a penalty to achieve the selection of significant features as well as the estimation of corresponding parameters. The lasso method preferably overcomes the insufficiencies of the traditional selection methods in the feature selection model.

Given the above, this study proposes a feature selection method (LAPLS) that unites the concepts of the lasso method and partial least squares (PLS). This method utilizes PLS to perform the regression and applies the L1 regular term constraint to the sum of the absolute values of the regression coefficients. In this way, the regression coefficients of insignificant features are compressed to 0, helping in achieving the goal of feature selection. This technique is then combined with the coordinate descent method to perform multiple iterations in order to select a higher-quality feature subset, which can then be used to screen the target for the “Gold Standard.” The new model algorithm not only can effectively overcome the issues of multicollinearity but can also eliminate insignificant features, making it suitable for the data analysis of traditional Chinese medicine.

## 3. Feature Selection Method Based on Partial Least Squares (LAPLS)

The lasso method uses L1 norm penalty regression to find the optimal solution [25, 26], in which a sparse weight matrix is generated that can be applied to feature selection. The basic idea is that under the constraint that the sum of the absolute values of the regression coefficients is less than or equal to a threshold *s* (i.e., ∑|*w*| ≤ *s*), the sum of the residual sums is minimized, so that the regression coefficients with smaller absolute values are compressed to 0. Following this, feature selection and corresponding parameter estimation are simultaneously realized, thus achieving an improved data dimensionality reduction effect.

The partial least squares method is a regression technique for solving multiple independent variables and multiple dependent variables. Compared with traditional regression analysis, PLS overcomes the problems of multicollinearity, small sample size, and variable number limit [27, 28]. For achieving the effective modeling purposes of partial least squares, firstly, extracting the latent variables *t*_1_ and *u*_1_ from the original independent variable *X* and the dependent variable *Y*, respectively, and simultaneously meet the criteria that *t*_1_ and *u*_1_ should represent the datasets as well as possible, and *t*_1_ has a strong explanatory power for *u*_1_ [29, 30]. If the accuracy condition is not met, the residual information is used to continue to carry out the second latent variables extraction until the accuracy condition is satisfied [31]. In the process of regression, however, the PLS method does not have the function of feature selection and cannot achieve an improved dimensionality reduction effect. Exploiting the lasso method, however, can effectively remove irrelevant and partially redundant features, thereby selecting better feature subsets. This study uses the lasso algorithm to optimize the partial least squares method, not only to achieve the feature selection functionality of partial least squares but also to achieve the screening of the “Gold Standard” via iteration. At the same time, this research helps us to overcome the problem of multicollinearity, which is an issue with the traditional lasso method.

**Figure 1:**
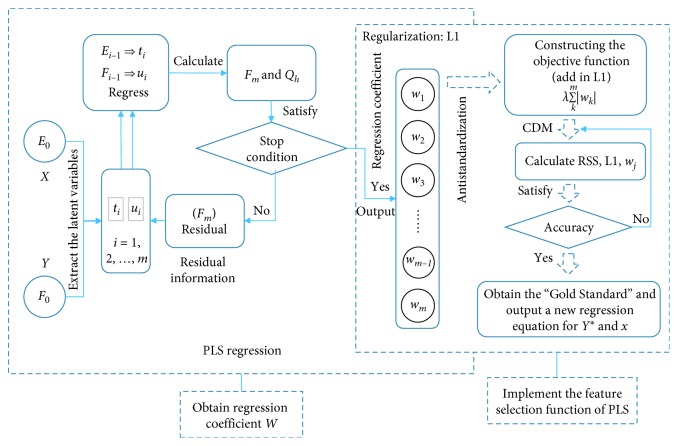
LAPLS structure.

The LAPLS method first uses the latent variables extracted by principal component analysis and canonical correlation analysis as the input for the multiple linear regression in partial least squares and then makes the sum of the absolute values of the coefficients less than or equal to a constant when performing the regression. That is to say, the L1 regular term constraint regression coefficient is added to the objective function, and, simultaneously, the sum of the squares of the residuals is minimized so that some regression coefficients strictly equal to 0 can be generated, thereby eliminating the irrelevant and partially redundant features. Combination with the coordinate descent method (CDM) [32] at last yields the “Gold Standard” feature subset. The construction process is shown in Figure 1.

The specific construction process is as follows:

 
*Step 1*. Standardize the data (*z*-score): *X*⇒*E*_0_, *Y*⇒*F*_0_.
 
*Step 2*. Extract the latent variables: the first latent variables *t*_1_ and *u*_1_ are, respectively, extracted from *E*_0_ and *F*_0_, with the goal of deriving the largest mutation information,var(*t*_1_)⟶max,  var(*u*_1_)⟶max; the largest degree of correlation, *r*(*t*_1_, *u*_1_)⟶max; and the largest comprehensive covariance of both sides, Cov(*t*_1_, *u*_1_)⟶max, in which *o*_1_, *c*_1_ is the first unit vector of *E*_0_, *F*_0_. *o*_1_, *c*_1_ can be obtained by solving the maximum value of *o*_1_^*T*^*E*_0_^*T*^*F*_0_*c*_1_, that is, using the Lagrangian algorithm; *o*_1_, *c*_1_ is the eigenvector corresponding to the largest eigenvalue of *E*_0_^*T*^*F*_0_*F*_0_^*T*^*E*_0_, *F*_0_^*T*^*E*_0_*E*_0_^*T*^*F*_0_. In this way, calculation of *t*_1_, *u*_1_ and the residual information matrix *E*_1_, *F*_1_ can be obtained, wherein *t*_1_ = *E*_0_*o*_1_, *u*_1_ = *F*_0_*c*_1_, *F*_1_ = *F*_0_ − *t*_1_*r*_1_^*T*^, *E*_1_ = *E*_0_ − *t*_1_*p*_1_^*T*^, *r*_1_ = (*F*_0_^*T*^*t*_1_)/‖*t*_1_‖^2^, and *p*_1_ = (*E*_0_^*T*^*t*_1_)/‖*t*_1_‖^2^. The residual information matrix *E*_1_, *F*_1_ is then substituted for *E*_0_, *F*_0_, after which *o*_2_, *c*_2_ and the second latent variables *t*_2_, *u*_2_ are then calculated according to the above steps.
 
*Step 3*. Judge whether satisfactory accuracy has been reached: according to the definition of cross-validity (1), if the currently extracted latent variables *t*_2_ makes the square of *Q*_*h*_ less than 0.0975 [30], then the added latent variables *t*_2_ has no significant effect on the prediction deviation of the reduction equation. Therefore, the previously extracted latent variable *t*_1_ is sufficient to achieve satisfactory accuracy, and the algorithm can be terminated. If, however, the currently extracted latent variable *t*_2_ makes the square of *Q*_*h*_ greater than 0.0975, and it can be assumed that increasing the latent variables *t*_2_ will improve the prediction accuracy. Therefore, the next latent variables are then extracted, and a judgment is made as to whether it is beneficial for reducing the prediction deviation of the equation. This process is cyclically looped until the satisfactory accuracy is no longer improved, and the algorithm can be terminated:

(1) $$\begin{array}{c} Q_{h}^{2}=1-\frac{\sum_{i=1}^{q}{\left(F_{0i}-F_{0h\left(-i\right)}\right)}^{2}}{\sum_{i=1}^{q}{\left(F_{0i}-F_{0\left(h-1\right)i}\right)}^{2}}, \end{array}$$

  where *q* is the number of samples; *h* is the latent variables number (*h* = 2,…, *r*); and *r* is the rank of the matrix *E*_0_; and *F*_0*h*(−*i*)_ is the fitting value of *F*_0_ at the sample point *i*, and the solution process is as follows: firstly, divide all sample points into two parts (one part including *n* − 1 and another part including one), and then use *n* − 1 sample points and the *h* latent variables for doing a regression equation, and finally the one sample point is substituted into the equation to obtain the fitted value *F*_0*h*(−*i*)_. In addition, all sample points are used to fit the regression equation containing *h* − 1 latent variables, and then the predicted value *F*_0(*h* − 1)*i*_ of the *i*th sample point can be obtained.
 
*Step 4*. Acquire the regression coefficients: assuming that the number of the latent variables extracted is *m* (*m* < *r*) when the satisfactory accuracy is achieved, the *t* regression equation for *F*_0_ and the inverse normalized equation are as shown in equation (2), thereby obtaining the regression coefficient *W* = (*w*_1_, *w*_2_,…, *w*_*m*_) that will be processed:

(2) $$\begin{array}{c} \left\{\begin{array}{c} F_{0}=t_{1}r_{1}^{T}+t_{2}r_{2}^{T}+\cdots +t_{m}r_{m}^{T}+F_{m},\\ Y=\sum w_{k}x_{k}+F_{ml}\;\left(k=1,2,\ldots ,m\right), \end{array}\right. \end{array}$$

  where *F*_*ml*_ is the *l* column of the residual matrix *F*_*m*_; *l* = 1,2,…, *L*; *L* is the number of dependent variables.
 
*Step 5*. Construct the objective function: after generating the coefficients using the above PLS regression, the function *J*(*w*) can be constructed by combining the regression coefficient *W* with the L1 regularization term of the lasso algorithm, in which the function satisfies the constraint that the sum of the absolute values of the regression coefficients *w*_*j*_ is less than or equal to a threshold. Under this condition, the sum of the squared residuals is minimized:

(3) $$\begin{array}{c} J\left(w\right)=\overset{q}{\underset{i=1}{\sum }}{\left(y_{i}-\overset{m}{\underset{j=1}{\sum }}w_{j}x_{j}\right)}^{2}+\lambda \overset{m}{\underset{j=1}{\sum }}\left|w_{j}\right|. \end{array}$$

  Minimization of the residual sum of squares:

(4) $$\begin{array}{c} \begin{array}{cc} \arg\underset{w}{\min} & \left\{\overset{q}{\underset{i=1}{\sum }}{\left(y_{i}-\overset{m}{\underset{j=1}{\sum }}w_{ij}x_{ij}\right)}^{2}\right\}\\ \text{s}.\text{t} & \overset{m}{\underset{j=1}{\sum }}\left|w_{j}\right|\leq s, \end{array} \end{array}$$

  where *q* is the number of samples; *s* is the threshold; and the parameter value is *λ* = *e*^(iter − *k*)^,  *k* ∈ [8,17].
 
*Step 6*. Solve the function: since the new model imposes the L1 regular term constraint on the regression coefficients generated by PLS, the resulting constructed function has absolute values, which makes the function underivable at the zero point. Therefore, this study uses the coordinate descent method [33] to solve the problem. It is worth noting that the new algorithm (LAPLS) is a reimplementation of the standard coordinate descent algorithm for lasso regression with an initial solution generated using PLS (Algorithm 1).
  First, the function is divided into 2 parts, RSS = ∑_*i*=1_^*q*^(*y*_*i*_ − ∑_*j*=1_^*m*^*w*_*j*_*x*_*j*_)^2^ and L1 = *λ*∑_*j*=1_^*m*^|*w*_*j*_|. Next, the partial derivative is calculated separately so that the overall partial derivative can be obtained:

(5) $$\begin{array}{c} \frac{\partial J\left(w\right)}{\partial w}=\left\{\begin{array}{cc} 2z_{j}w_{j}-2\rho_{j}-\lambda , & w_{j}<0,\\ \left[-2\rho_{j}-\lambda ,-2\rho_{j}+\lambda \right], & w_{j}=0,\\ 2z_{j}w_{j}-2\rho_{j}+\lambda , & w_{j}>0, \end{array}\right. \end{array}$$

  where *ρ*_*j*_ = ∑_*i*=1_^*q*^*x*_*j*_(*y*_*i*_ − ∑_*k*≠*j*_*w*_*k*_*x*_*k*_), *z*_*j*_ = ∑_*i*=1_^*q*^*x*_*i*_^2^.
  Let ∂*j*(*w*)/∂*w* = 0, and then determine the regression coefficient *w*_*j*_ under the regular term constraint:

(6) $$\begin{array}{c} w_{j}=\left\{\begin{array}{cc} \frac{\left(\rho_{j}+\lambda /2\right)}{z_{j}}, & \rho_{j}<\left(-\frac{\lambda }{2}\right),\\ 0, & \rho_{j}\in \left(\left(-\frac{\lambda }{2}\right),\frac{\lambda }{2}\right),\\ \frac{\left(\rho_{j}-\lambda /2\right)}{z_{j}}, & \rho_{j}>\frac{\lambda }{2}. \end{array}\right. \end{array}$$

 
*Step 7*. Perform multiple iterations through Step 6, in which some regression coefficients are strictly compressed to 0 at a certain iteration so that the best feature subset (i.e., the “Gold Standard”) can be selected. The selected subset of features is then regressed to construct a simpler optimization model than PLS regression:

(7) $$\begin{array}{c} Y^{*}=\sum w_{h}^{*}x_{h}+F_{h}^{*},\;h<m. \end{array}$$

## 4. Experimental Design

### 4.1. Experimental Data Description

In this study, the 6 experimental datasets included the traditional Chinese medicine data (WYHXB, NYWZ, and DCQT) from the Key Laboratory of Modern Chinese Medicine Preparations Ministry of Education as well as Communities and Crime (CCrime), Breast Cancer Wisconsin (Prognostic) (BreastData), and Residential Building Dataset (RBuild) from the UCI Machine Learning Repository. The basic information for each dataset is listed in Table 1. There are 798 features in WYHXB, 1 dependent variable, and 54 samples; 10283 features in NYWZ, 1 dependent variable, and 54 samples; 9 features in DCQT, 1 dependent variable, and 10 samples; CCrime describes community crime and includes 127 features, 1 dependent variable, and 1994 samples; BreastData describes breast cancer cases and includes 34 features, 1 dependent variable, and 198 samples; and RBuild describes residential buildings and includes 103 features, 1 dependent variable, and 372 samples. Since the UCI datasets obtained from the UCI Machine Learning Repository generally had numerous missing values, the mean filling method was used for data preprocessing during the experiment. The reason this study adopted CCrime, BreastData, and RBuild from the UCI datasets for the experiment was to compare the regression effects of the new model on a public dataset consisting of diversified data, in order to validate the reliability and robustness of the new model.

WYHXB and NYWZ are the basic experimental data of Shenfu injections used to treat cardiogenic shock, which utilizes the left anterior descending coronary artery near the edge of the heart to replicate the middle-end cardiogenic shock rat model. In seven groups of 6 shock model rats, each were injected with 0.1, 0.33, 1.0, 3.3, 10, 15, or 20 mL·kg^−1^ of Shenfu. There was also a model setting group and a blank group. Sixty minutes after receiving the Shenfu, the red blood cell flow rate (*μ* m/s) pharmacodynamic indicator was collected. The material information contained in the Shenfu injection is called the exogenous substance (i.e., the WYHXB data, as shown in Table 2), and the material information of the experimental individual is called the endogenous substance (i.e., the NYWZ data, as shown in Table 3). In the 2 datasets, the material information is the independent variable (i.e., the feature), and the red blood cell flow rate is the dependent variable.

The experimental data of the traditional Chinese medicine (DCQT) were mainly used to study the factors affecting the physiological index (d-lactic acid content) under the influence of the active ingredients in Chinese medicinal rhubarb. The contents (characteristics) of the active ingredients of the rhubarb include aloe emodin, emodin, rhein and chrysophanol, emodin methyl ether, magnolol, as well as honokiol, hesperidin, and synephrine. The dependent variable is D(−)-lactic acid content. The partial experimental data are listed in Table 4.

### 4.2. Results and Discussion

#### 4.2.1. Experimental Parameters

Using a strategy of selecting and optimizing the parameters corresponding to each dataset with the goal of ensuring model result reliability is significant given that the experimental data themselves have different characteristics and the corresponding model parameters are inconsistent. First, the model parameters were initialized and set to *s*=0.1 and *λ*=*e*^(iter − 10)^, where *s* is the model threshold, *λ* is the model parameter value, and iter is the number of iterations. Next, based on the initialization value, a comparison strategy was used for analysis. Specifically, with the maintenance condition of *s*=0.1, the *λ* value was gradually increased or decreased until the best *λ* value was determined based on the R-squared evaluation index (as shown in Table 5). Finally, by keeping the selected *λ* value for each dataset fixed while increasing or decreasing the threshold *s*, a set of model parameters could preferentially be selected (as shown in Table 6).

In Table 5, keeping *s*=0.1 fixed, the results are as follows: for the WYHXB data, the result of the homologous R-squared evaluation indicates that the best result occurs when *k* = 13 (i.e., *λ*=*e*^(iter − 13)^); for the NYWZ data, the best result occurs when *k* = 12 (i.e., *λ*=*e*^(iter − 12)^); for the DCQT data, the best result occurs when *k* = 10 (i.e., *λ*=*e*^(iter − 10)^); for the CCrime data, the best result occurs when *k* = 12 (i.e., *λ*=*e*^(iter − 12)^); for the BreastData data, the best result occurs when *k* = 12 (i.e., *λ*=*e*^(iter − 12)^); and for the RBuild data, the best result occurs when *k* = 13 (i.e., *λ*=*e*^(iter − 13)^). By comparing the results of Table 6, one group of optimal parameters for each dataset can be selected: for the WYHXB data, we should choose *λ*=*e*^(iter − 13)^, *s*=0.1; for the NYWZ data, we should choose *λ*=*e*^(iter − 12)^, *s*=0.11; for the DCQT data, we should choose *λ*=*e*^(iter − 10)^, *s*=0.1; for the CCrime data, we should choose *λ*=*e*^(iter − 12)^, *s*=0.11; for the BreastData data, we should choose *λ*=*e*^(iter − 12)^, *s*=0.13; and for the RBuild data, we should choose *λ*=*e*^(iter − 13)^, *s*=0.05.

**Figure 2:**
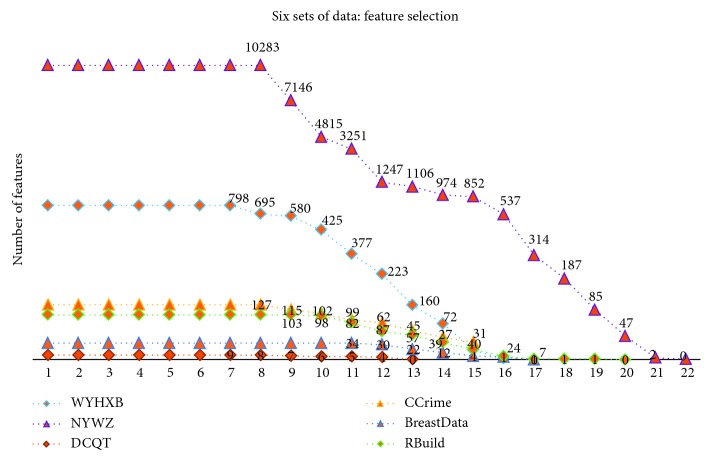
Change in the number of features.

**Figure 3:**
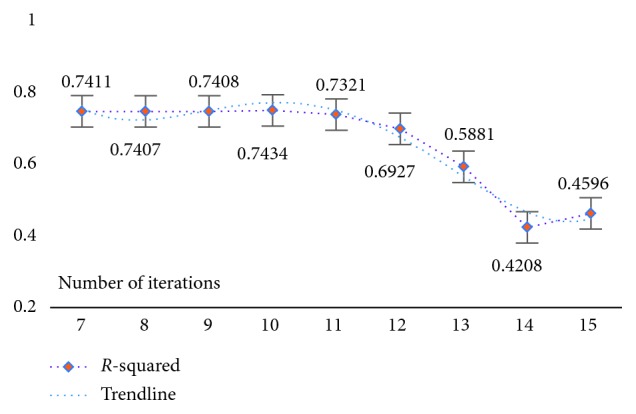
WYHXB: *R*-squared.

**Figure 4:**
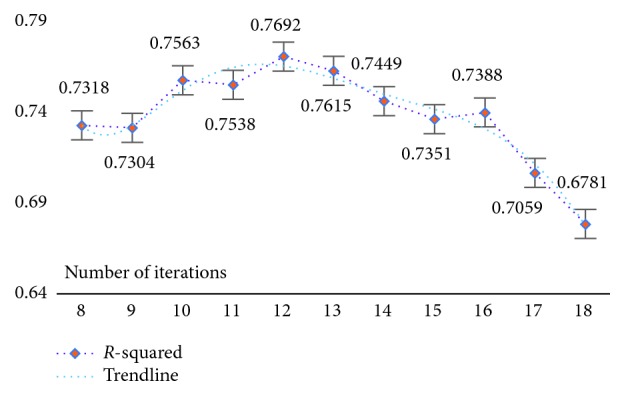
NYWZ: *R*-squared.

**Figure 5:**
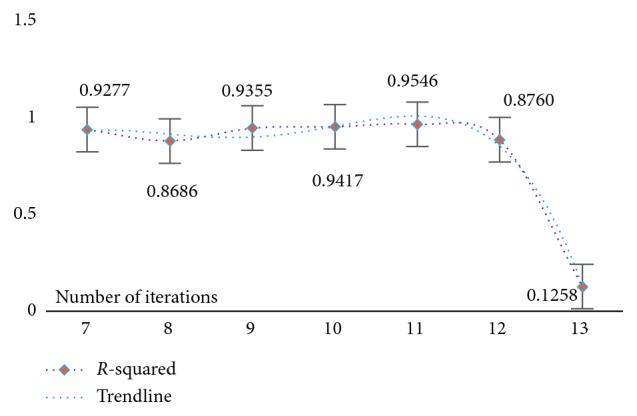
DCQT: *R*-squared.

**Figure 6:**
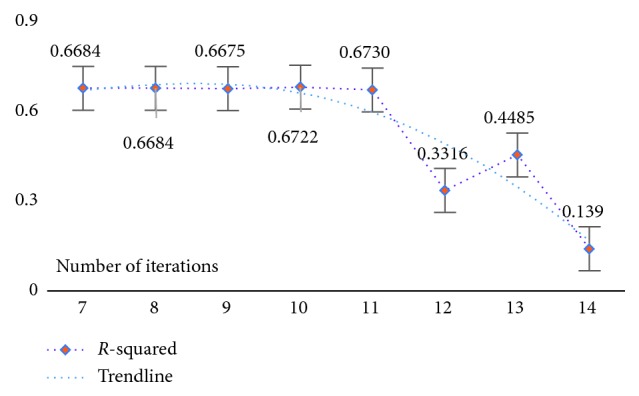
CCrime: *R*-squared.

**Figure 7:**
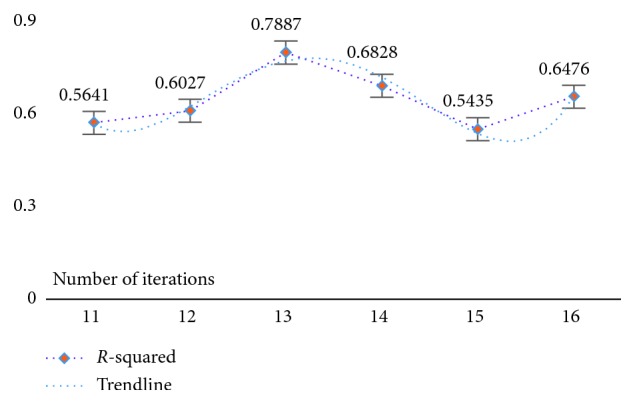
BreastData: *R*-squared.

**Figure 8:**
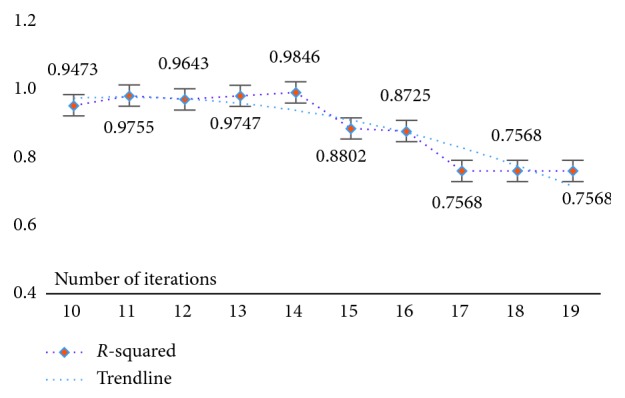
RBuild: *R*-squared.

At the same time, in order to verify the feasibility and availability of the LAPLS method, we should consider the feature analyses of each dataset (with the model parameters utilizing the above results) during our experimentation. Specifically, by determining the iteration at a particular time (the number of iterations ranged from 1 to 25), the model achieving the best feature subset (“Gold Standard”) and the best homologous R-squared value can be selected. The results of this analysis are shown in Figures 2–8. It can be seen in Figure 2 that the number of features in the 6 groups of experimental data decreased as the number of iterations increased, indicating the trend towards the goal of eliminating irrelevant and partially redundant features. Similarly, Figures 3–8 show the trend of the corresponding R-squared values during the iterative process of each dataset (i.e., the stages as the number of features change). This does not indicate, however, that the fewer the number of features, the better the calculated result. The specific results were as follows: for the WYHXB data, after 10 iterations, 425 significant features could be selected (373 features had been eliminated), and the homologous R-squared value was optimal; for the NYWZ data, after 12 iterations, 1247 significant features could be selected (9036 features had been eliminated) and the homologous R-squared value was optimal; for the DCQT data, after 11 iterations, 5 significant features could be selected (4 features had been eliminated) and the homologous R-squared value was optimal; for the CCrime data, after 11 iterations, 82 significant features could be selected (45 features had been eliminated) and the homologous R-squared value was optimal; for the BreastData data, after 13 iterations, 22 significant features could be selected (12 features had been eliminated) and the homologous R-squared value was optimal; for the RBuild data, after 14 iterations, 39 significant features could be selected (64 features had been eliminated) and the homologous R-squared value was optimal.

**Figure 9:**
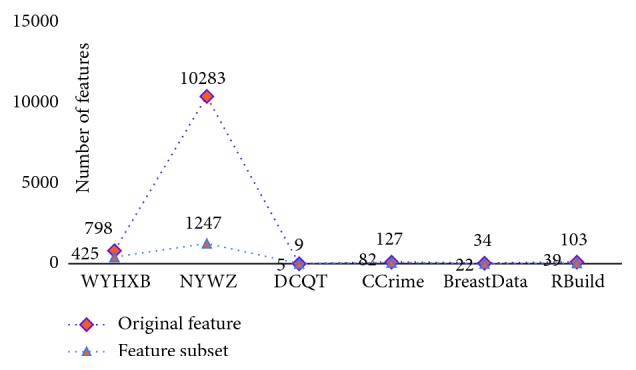
Feature selection results for the 6 datasets.

From the above experiments, we could determine the respective parameters and the homologous iteration times for each of the 6 datasets and also obtain the dimensionality reduction effect of each feature selection by the new model (i.e., the degree of irrelevant and redundant feature elimination), as shown in Figure 9. It is worth noting, however, that the number of eliminated features cannot be 0, given the meaning of feature selection.

#### 4.2.2. Comparison of the LAPLS with Other Methods

Further analysis of the new model consisted of randomly dividing each dataset into a training set and a test set (ratio = 7 : 3) and then utilizing traditional partial least squares (PLS), lasso, PLSRFE, and the improved algorithm (LAPLS) for training and learning. The test set was subjected to a regression experiment (with parameters and number of iterations consistent with the previously listed optimal values), in which the *R*-squared (*R*^2^) and root-mean-square error (RMSE) values were used as the model evaluation indicators. Meanwhile, in order to ensure the reliability of the experimental results, 10 tests were performed for each set of experimental data, and ultimately, the respective average values were chosen as the final experimental results, as shown in Table 7. Via the above experimental design, the verification of the new model could be analyzed from two perspectives: (1) the comparison between the LAPLS and the traditional methods (PLS, lasso) and (2) the comparison between the LAPLS and the same type of feature selection method (PLSRFE).

Table 7 lists the experimental results of LAPLS regression on the test sets of 6 sets of original data (WYHXB, NYWZ, DCQT, CCrime, BreastData, and RBuild). The *R*^2^ values were 0.6558, 0.7326, 0.9384, 0.6703, 0.7064, and 0.9831, respectively, and the RMSE were 412.7325, 140.5172, 0.0117, 0.1516, 3.1468, and 202.5260, respectively. Compared with PLS and lasso, the LAPLS had a slightly inferior RMSE for the CCrime (larger sample size) data (0.0128 greater error than PLS and 0.0212 greater error than lasso), but for the results of the remaining experimental datasets, the new method performed better than the traditional methods. Compared with the PLSRFE, the LAPLS was slightly inferior for the CCrime data and the LAPLS RMSE was slightly higher than the PLSRFE RMSE for the RBuild data, although the LAPLS results were better than those of the PLSRFE for the remaining experimental data. Overall, the results of the improved algorithm were better than those of the other existing algorithms, indicating that the new model has the effect of eliminating irrelevant and redundant features. In addition, as shown by the experimental results, the new model proved to be relatively adaptable, demonstrating effectiveness not only for multifeature data but also for data with fewer features.

**Figure 10:**
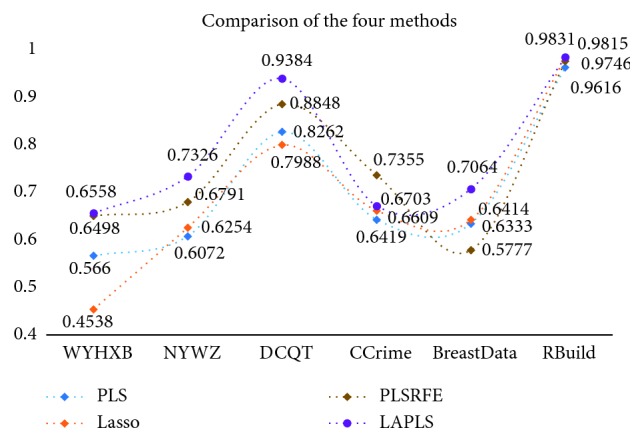
Experimental results of 6 datasets (*R*-squared).

**Figure 11:**
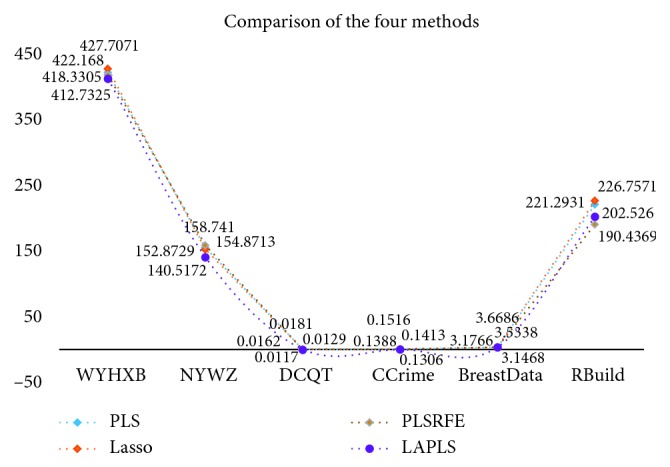
Experimental results of 6 datasets (RMSE).

In order to observe the experimental results more intuitively, the trend graphs were portrayed separately (Figures 10 and 11) in order to reflect the fluctuations of the *R*^2^ and RMSE values. It can be seen that the *R*^2^ and RMSE values of the new model for the 6 sets of experimental data are basically superior to those of the other algorithms, indicating that the regression results of the new model are better and that this approach effectively eliminates the irrelevant and partially redundant features. In summary, the improved algorithm not only performs feature selection and screens out the “Gold Standard” feature subset for general high-dimensional data but also could well suit the traditional Chinese medicine data.

## 5. Conclusions

The traditional partial least squares method has no feature selection function, and the goal of obtaining of a higher-quality feature subset cannot be achieved for experimental data from traditional Chinese medicine. Given this, we proposed a feature selection method based on partial least squares. This method made sufficient use of the advantages of the lasso algorithm, namely, imposing constraints on the sum of the absolute values of the regression coefficients and carrying out feature selection, while simultaneously combining this technique with the partial least squares method, which can solve the multicollinearity problem in order to perform regression analysis. In this way, both data dimensionality reduction and the screening of the “Gold Standard” feature subset were realized. Through the experimental comparison of TCM data and UCI datasets, it was clearly demonstrated that the improved algorithm significantly strengthens the interpretation degree and prediction accuracy of the model, and it is a suitable analytical method for TCM data. The improved algorithm, however, has the disadvantage of only eliminating the partially redundant features from high-dimensional data. Going forward, we will continue to improve the algorithm in order to boost its efficiency. In addition, ensuring that reasonable relevant parameters are set during model creation also requires further study.

## Figures and Tables

**Algorithm 1:**
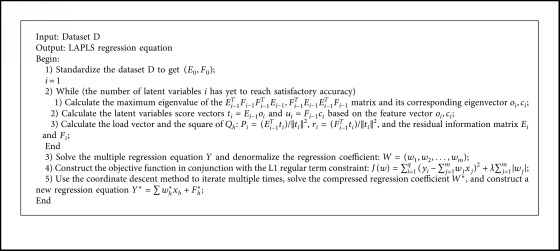
LAPLS

**Table 1:** Basic dataset information (default task: regression).

Dataset	Number of samples	Number of attributes
WYHXB	54	799 (798 + 1)
NYWZ	54	10284 (10283 + 1)
DCQT	10	10 (9 + 1)
CCrime	1994	128 (127 + 1)
BreastData	198	35 (34 + 1)
RBuild	372	104 (103 + 1)

**Table 2:** Partial data of basic experiments with traditional Chinese medicine substances (WYHXB).

0.34_237.0119 (m/z)	0.35_735.1196 (m/z)	0.36_588.0942 (m/z)	…	0.36_590.0903 (m/z)	Red blood cell flow rate (*μ* m/s)
0.48808	302.16	0	…	27.8589	750
100.078	62.016	0	…	3.80712	1400
11.6992	52.5058	7.61005	…	4.85059	785
143.643	284.113	0	…	456.607	790
7.75089	54.4535	0	…	0	670
18.2499	0	0	…	14.6621	680
…	…	…	…	…	…
28.5783	0	0	…	2.3551	850
2.91064	0	16.1624	…	3.41406	620
…	…	…	…	…	…

**Table 3:** Partial data of basic experiments with traditional Chinese medicine substances (NYWZ).

11.10_787.5077 (m/z)	12.29_526.1784 (m/z)	12.29_531.2005 (m/z)	…	12.47_631.3847 (m/z)	Red blood cell flow rate (*μ* m/s)
53.3719	11557.6	764.329	…	1795.79	2200
43.4717	7971.33	875.465	…	1842.39	2750
76.507	3399.9	870.161	…	1562.81	1980
153.145	51027.4	916.064	…	1619.62	1860
16.3197	10694.4	942.699	…	1612.42	2100
42.2836	11048.1	714.536	…	1649.23	2000
…	…	…	…	…	…
55.5021	4702.83	748.844	…	1632.9	2481
153.21	78912.8	835.24	…	1647.55	2970
…	…	…	…	…	…

**Table 4:** Effects of active ingredients in traditional Chinese medicine on physiological indices (DCQT).

Aloe emodin	Emodin	Rhein	…	Synephrine	D(−)-lactic acid
0.0625	0.0468	0.0945	…	0.2198	0.0625
0.0450	0.0317	0.0558	…	0.4865	0.0525
0.0075	0.0085	0.0126	…	0.0176	0.0300
0.0350	0.0278	0.0434	…	0.0709	0.0400
…	…	…	…	…	…
0.1006	0.0875	0.1841	…	0.1239	0.0575
0.1060	0.0960	0.1982	…	0.0536	0.1325
0.0540	0.0441	0.0871	…	0.0471	0.1900

**Table 5:** R-squared (*s*=0.1 constant) for 6 datasets with different *λ*=*e*^(iter − *k*)^ values.

	*k* = 8	*k* = 9	*k* = 10	*k* = 11	*k* = 12	*k* = 13	*k* = 14	*k* = 15	*k* = 16	*k* = 17
WYHXB	0.4756	0.5412	0.5881	0.6927	0.7321	**0.7434**	0.7408	0.7407	0.7411	0.7411
NYWZ	0.5827	0.6938	0.6521	0.7434	**0.7689**	0.7615	0.7452	0.7316	0.7294	0.7294
DCQT	0.1258	0.8760	**0.9546**	0.9417	0.9355	0.8686	0.9277	0.9277	0.9277	0.9277
CCrime	0.4517	0.4517	0.5320	0.6621	**0.6708**	0.6680	0.6684	0.6684	0.6684	0.6684
BreastData	0.6475	0.6475	0.5435	0.6829	**0.7436**	0.5697	0.5728	0.5732	0.5641	0.5641
RBuild	0.7567	0.7567	0.7567	0.8195	0.8801	**0.9593**	0.9404	0.9181	0.9045	0.8757

**Table 6:** Comparative analysis of several parameter combinations for 6 datasets.

*s*, *λ*=*e*^(iter − *k*)^	*R* ^2^
*WYHXB*	
*s* = 0.001, *k* = 13	0.7366
*s* = 0.005, *k* = 13	0.7366
*s* = 0.01, *k* = 13	0.7427
*s* = 0.05, *k* = 13	0.7403
**s** **=** **0.1, k** **=** **13**	**0.7434**
*s* = 0.11, *k* = 13	0.7434
*s* = 0.12, *k* = 13	0.7434
*s* = 0.13, *k* = 13	0.7418
*s* = 0.14, *k* = 13	0.7418
*s* = 0.15, *k* = 13	0.7418
*s* = 0.20, *k* = 13	0.7235

*NYWZ*	
*s* = 0.001, *k* = 12	0.7361
*s* = 0.005, *k* = 12	0.7361
*s* = 0.01, *k* = 12	0.7435
*s* = 0.05, *k* = 12	0.7456
*s* = 0.1, *k* = 12	0.7689
**s** **=** **0.11, k** **=** **12**	**0.7692**
*s* = 0.12, *k* = 12	0.7692
*s* = 0.13, *k* = 12	0.7468
*s* = 0.14, *k* = 12	0.7468
*s* = 0.15, *k* = 12	0.7468
*s* = 0.20, *k* = 12	0.7344

*DCQT*	
*s* = 0.001, *k* = 10	0.8760
*s* = 0.005, *k* = 10	0.8760
*s* = 0.01, *k* = 10	0.8760
*s* = 0.05, *k* = 10	0.8760
**s** **=** **0.1, k** **=** **10**	**0.9546**
*s* = 0.11, *k* = 10	0.9546
*s* = 0.12, *k* = 10	0.9546
*s* = 0.13, *k* = 10	0.9546
*s* = 0.14, *k* = 10	0.9546
*s* = 0.15, *k* = 10	0.9546
*s* = 0.20, *k* = 10	0.9546

*CCrime*	
*s* = 0.001, *k* = 12	0.6524
*s* = 0.005, *k* = 12	0.6524
**s** **=** **0.01, k** **=** **12**	**0.6722**
*s* = 0.05, *k* = 12	0.6625
*s* = 0.1, *k* = 12	0.6708
*s* = 0.11, *k* = 12	0.6620
*s* = 0.12, *k* = 12	0.6678
*s* = 0.13, *k* = 12	0.6678
*s* = 0.14, *k* = 12	0.6678
*s* = 0.15, *k* = 12	0.6678
*s* = 0.20, *k* = 12	0.6678

*BreastData*	
*s* = 0.001, *k* = 12	0.6419
*s* = 0.005, *k* = 12	0.6419
*s* = 0.01, *k* = 12	0.6419
*s* = 0.05, *k* = 12	0.7401
*s* = 0.1, *k* = 12	0.7436
*s* = 0.11, *k* = 12	0.7436
*s* = 0.12, *k* = 12	0.7436
**s** **=** **0.13, k** **=** **12**	**0.7887**
*s* = 0.14, *k* = 12	0.7887
*s* = 0.15, *k* = 12	0.7887
*s* = 0.20, *k* = 12	0.7213

*RBuild*	
*s* = 0.001, *k* = 13	0.9407
*s* = 0.005, *k* = 13	0.9407
*s* = 0.01, *k* = 13	0.9589
**s** **=** **0.05, k** **=** **13**	**0.9845**
*s* = 0.1, *k* = 13	0.9593
*s* = 0.11, *k* = 13	0.9593
*s* = 0.12, *k* = 13	0.8803
*s* = 0.13, *k* = 13	0.8803
*s* = 0.14, *k* = 13	0.8803
*s* = 0.15, *k* = 13	0.9285
*s* = 0.20, *k* = 13	0.9285

**Table 7:** Comparison of experimental results of the LAPLS with other methods (evaluation indicators: *R*^2^ and RMSE).

	PLS		Lasso		PLSRFE		LAPLS	
	*R* ^2^	RMSE	*R* ^2^	RMSE	*R* ^2^	RMSE	*R* ^2^	RMSE
WYHXB	0.5660	422.1680	0.4538	427.7071	0.6498	418.3305	**0.6558**	**412.7325**
NYWZ	0.6072	154.8713	0.6254	152.8729	0.6791	158.7410	**0.7326**	**140.5172**
DCQT	0.8262	0.01620	0.7988	0.0129	0.8848	0.0181	**0.9384**	**0.0117**
CCrime	0.6419	0.1388	0.6609	**0.1306**	**0.7355**	0.1413	0.6703	0.1516
BreastData	0.6333	3.5338	0.6414	3.1766	0.5777	3.6686	**0.7064**	**3.1468**
RBuild	0.9616	221.2931	0.9815	226.7571	0.9746	1**90.4369**	**0.9831**	202.5260
Average	0.7060	133.6702	0.6936	135.1095	0.7502	128.5561	**0.7811**	**126.5143**

## Data Availability

The TCM data used in this study can be obtained by contacting the first author, and other data sets can be obtained through the UCI Machine Learning Repository.
